# The Tale of CHD4 in DNA Damage Response and Chemotherapeutic Response

**Published:** 2019-07-08

**Authors:** Jing Zhang, David J.H. Shih, Shiaw-Yih Lin

**Affiliations:** 1 Department of Systems Biology, The University of Texas MD Anderson Cancer Center, Houston, Texas 77030, USA.

**Keywords:** CHD4, DNA damage response, mutations, chemotherapeutic response

## Abstract

The chromatin remodeling factor chromodomain helicase DNA-binding protein 4 (CHD4) is a core component of the nucleosome remodeling and deacetylase (NuRD) complex. Due to its important role in DNA damage repair, CHD4 has been identified as a key determinant in cancer progression, stem cell differentiation, and T cell and B cell development. Accumulating evidence has revealed that CHD4 can function in NuRD dependent and independent manner in response to DNA damage. Mutations of CHD4 have been shown to diminish its functions, which indicates that interpretation of its mutations may provide tangible benefit for patients. The expression of CHD4 play a dual role in sensitizing cancer cells to chemotherapeutic agents, which provides new insights into the contribution of CHD4 to tumor biology and new therapeutic avenues.

## Introduction

Known as Mi2β, Chromodomain helicase DNA-binding 4 (CHD4), a highly conserved protein (~250 kDa) in animals and plants [1], is one of members of the SNF2/RAD54 helicase family, which use the energy derived from ATP hydrolysis to remodel nucleosome structure [2, 3]. It comprises a core ATPase/helicase domain flanked by two Plant Homeodomain motifs (PHD fingers) that recognize modifications of histone tails, tandem chromodomains, and carboxyl- terminal domains necessary for transcriptional repression.

The ATPase domain enables CHD4 to use energy from ATP hydrolysis to remodel nucleosomes along DNA [4]. In this context, CHD4 serves as a key component of the nucleosome remodeling and deacetylase (NuRD) complex, which plays an important role in regulation of chromatin structure, gene expression, and cell cycle progression during normal development and tumorigenesis [5, 6]. In addition to CHD4, NuRD also contains a core of two lysine deacetylase proteins, HDAC1 and HDAC2, two chaperone proteins, RBBP4 and RBBP7, together with associated proteins [1, 7, 8]. It is important to point out that CHD4-NuRD complex is recruited to sites by interaction with sequence-specific DNA binding proteins, instead of recognizing specific DNA sequences [9–11]. After the interaction with DNA binding protein, the local chromatin structures are modified through nucleosomes remodeling, histone deacetylation, and promoter decommissioning [12–14]. Therefore, CHD4-NuRD complex has been identified as an integral driver of transcription.

Although CHD4 is defined as core NuRD component, increasing evidence is accumulating that CHD4 also play important NuRD-independent role in the DNA-damage response, cell cycle progression, signal transduction, and overall genome maintenance [8, 15, 16]. An early study shows that CHD4 associates with the CD4 enhancer independently of NuRD to activate CD4 expression during T-cell development[15]. In the Ikaros-deleted thymocyte system, CHD4 functions in a NuRD-independent manner to antagonize PRC2- mediated transcriptional repression [17]. In addition, the PHD fingers of CHD4 enable it to directly bind both un-methylated (H3K4) as well as K9-methylated (H3K9me3) N-terminal tails of histone H3 [18, 19]. The dual properties of CHD4 that both regulate chromatin structure via ATP hydrolysis and bind histone H3 indicate that it involves in different aspects of chromatin accessibility.

Despite the increasing characterization of this transcriptional regulator, we still know little about the function of CHD4 in cancer progression as a DNA-repair protein. Recent studies suggest the roles CHD4 in both promoting and suppressing tumor growth [20–24]. For instance, CHD4 expression level is positively correlated with the malignant progression of non-small-cell lung cancer [25]. More recently, CHD4 expression was found to be positively correlated with metastatic stage, tumor recurrence and survival status in triple-negative breast cancer (TNBC) [21]. Interestingly, CHD4 is also implicated as a tumor suppressor in some cancer types [22]. We previously reported that CHD4 depletion and CHD4 mutations promote endometrial cancer stemness by activating TGF-beta signaling [23]. In this review, we summarized the role of this transcriptional regulator in DNA repair and its potential roles in the development of novel anti-cancer drugs.

## The role of CHD4 in regulating DNA damage response

In response to DNA damage, cells initiate DNA-damage response (DDR) to prevent tumorigenesis [26–28]. The activation of DDR involves in the recruitment of DDR proteins to the damaged sites in an orchestrated manner. Increasing evidence has demonstrated that CHD4 plays an important role in genome integrity by regulating signaling and repair in response to DNA damage [29–31]. However, it is not clear whether CHD4 functions as a part of NuRD or independently of NuRD.

In response to DNA damage, CHD4 can be recruited to the sites of DNA damage by different models. One model is NuRD complex dependent, where CHD4-NuRD complex is rapidly recruited to the sites of DNA damage through the association with Poly(ADP-ribose) polymerase 1 (PARP1) [31–33]. In this model, CHD4 helps set up a transient repressive chromatin structure at sites of DNA damage to facilitate DNA repair. Another model is NuRD complex independent, where CHD4 is recruited to the sites of DNA damage by RING finger ubiquitin ligase 8 (RNF8), which promotes assembly of a subset of DNA repair factors such as RNF168 and BRCA1 at the sites of the DNA damage [16]. In addition, CHD4 can be phosphorylated by the DNA damage response kinases ATM [34, 35] and ATR [36], and in turn CHD4 can also regulate the Tip60- dependent phosphorylation of ATM [37] in response to DNA damage.

However, what is currently unknown is that whether CHD4 functions to maintain genome integrity prior to DNA damage, or it is required to repair damage upon DNA damage. For instance, CHD4 was reported to modulate the expression of RAD51, an essential protein in homologous recombination of DNA, by directly binding to the RAD51 promoter [30]. And loss of CHD4, even without the DNA damage agents, caused DNA damage in glioblastoma cells by decreasing the activity of RAD51 [30]. Interestingly, CHD4 was also shown to be recruited to DNA damage sites in poly(ADP-ribosyl)ation-dependent manner during chromatin remodeling in response to DNA damage induction [31]. Taken together, CHD4 appears to have a key role for DNA repair and cell survival through multiple mechanisms.

## CHD4 and its mutations in cancer progression

Cancers with CHD4 mutations showed loss of CHD4 expression. Loss of CHD4 expression was found in over 50% of gastric and colorectal cancers [38]. In endometrial cancer, CHD4 is one of the most frequently mutated tumor suppressor genes and typically sustains missense mutations (instead of deletions) [39, 40]. Despite the highly frequent mutations of CHD4 detected, the functional significance of most CHD4 mutations is unknown. We recently showed that two point mutations of CHD4 (R975H and R1162W) in ATPase domain in endometrial cancer destabilized the CHD4 protein and consequently diminished the function of CHD4, which induces a cancer stem cell phenotype to promote cancer progression [23]. Miki Yamada et al. reported that missense single nucleotide variation p.D140E accelerated the development of cancer [41]. Therefore, interpretation of mutations in CHD4 may provide tangible benefit for patients with cancer in the near term.

In addition to its role in cancer progression, CHD4 plays essential roles in stem cell differentiation [14, 42, 43], embryonic development [44, 45], striated muscle identity [46], and T cell [15] and B cell development [47, 48]. Through whole-exome sequencing and web-based gene matching, five individual de novo mutations in CHD4 were identified to cause an intellectual disability syndrome with distinctive dysmorphisms [49]. CHD4 depletion has been shown to sensitize cancer cells to some chemotherapeutic agents [23]. Therefore, these loss-of-function mutations show potential for therapeutic targeting. Together, it is important to identify CHD4 mutations that induce sensitivity to chemotherapeutic agents. A library of all clinically observed CHD4 mutations could be generated by high- throughput cloning in order to identify loss-of-function mutations that confer sensitivity to chemotherapeutic agents.

## The role of CHD4 in chemotherapeutic response

CHD4 expression was found to be associated with metastatic stage, tumor recurrence and survival status, and loss of CHD4 expression sensitizes cells to DNA-damaging agents [37, 50, 51]. We previously demonstrated the importance of CHD4 in controlling HR repair to maintain genome stability, and that deficiency in CHD4 impairs the recruitment of BRIT1 and sensitizes cells to poly (ADP-ribose) polymerase inhibitor treatment [51]. Subsequently, an siRNA screen by Carolian D’Alesio et al. identified CHD4 as a therapeutic target in colon cancer. In this study, CHD4 depletion was found to sensitize colon cancer cells to DNMT inhibitors in reactivating hypermethylated genes [52]. Later, depletion of CHD4 in acute myeloid leukemia blasts was found to sensitize cells both *in vitro* and *in vivo* to genotoxic agents daunorubicin and cytarabine [37]. More recently, CHD4 was found to mediate epithelial-mesenchymal transition in TNBC cells, and silencing of CHD4 expression in these cells increase drug sensitivity to cisplatin and PARP1 inhibitor [21].

Interestingly, CHD4 depletion in BRCA2 mutant cells promotes resistance to cisplatin by a mechanism independent of HR but dependent on RAD18 [53]. Loss of CHD4 in BRCA2 mutant PEO1 cells showed enhanced survival against the DNA-damaging agents such as the PARP inhibitor Olaparib, the double-strand break- inducing agent Zeocin, and the DNA polymerase inhibitor aphidicolin [53]. These seemingly contradictory results reveal the intricate mechanisms of CHD4 involved. To date, no therapeutic agents have been reported to exhibit selectivity towards the expression of CHD4.

Therefore, CHD4 may be serve as a potential target for the development of new anti-cancer agents.

## Conclusion and future directions

Recent progress in understanding the role of CHD4 in response to DNA damage, as discussed above, suggests the importance of designing effective cancer therapeutic agents that target CHD4. However, due to the crucial roles of CHD4 in both promoting and suppressing tumor growth, more knowledge of the fundamental biology downstream of CHD4 should be investigated. Identifying the binding partners of CHD4 and the signaling cascade its involved in will facilitate the generation of models revealing the biological functions of the CHD4 to cancer.

Loss-of-function mutations of CHD4 has been observed in some cancer types. Clinical genome-sequencing projects should be conducted to provide insight into the prevalence of CHD4 mutations in different cancer types and reveal patterns of these mutations. High-throughput screens of compounds associated with CHD4 and its mutations may provide new insights into the contribution of CHD4 aberrations to tumorigenesis and provide new therapeutic avenues.

In addition, the expression of CHD4 plays a dual role in sensitizing cancer cells to chemotherapeutic agents. This dual role mechanism is related to the role of CHD4 in DNA damage repair. However, it is not clear whether or not CHD4 drives resistance and how it does this. As CHD4 frequently associates with DDR proteins to regulate transcription, drugs regulating the activity or the interactions of these proteins may be a more selective approach to inhibiting undesirable CHD4 functions in cancer cells. Also post-translational modification of CHD4 can modulate its function, which offers potentially additional drug targets for novel cancer therapies.
